# The effect of breastfeeding on postpartum glucose tolerance and lipid profiles in women with gestational diabetes mellitus

**DOI:** 10.1186/s13006-019-0238-5

**Published:** 2019-11-04

**Authors:** Alexis Shub, Manisha Miranda, Harry M. Georgiou, Elizabeth A. McCarthy, Martha Lappas

**Affiliations:** 1 Department of Obstetrics and Gynecology, University of Melbourne, Parkville, Victoria Australia; 2 Perinatal Department, Mercy Hospital for Women, Heidelberg, Victoria Australia

**Keywords:** Gestational diabetes mellitus, Breastfeeding, Glucose intolerance

## Abstract

**Background:**

We aimed to investigate the association of breastfeeding on postpartum glucose levels and lipid profiles in women diagnosed with gestational diabetes mellitus (GDM) and women without GDM.

**Methods:**

We performed a secondary analysis of a cohort study of 243 women, 159 women with GDM and 84 normally glucose tolerant women between 2012 and 2017. At approximately 6–10 weeks postpartum, we measured fasting blood glucose and plasma lipid levels. Breastfeeding behaviour was self-defined as exclusive breastfeeding or not exclusive breastfeeding.

**Results:**

The mean (SD) glucose in the group of women who breastfed exclusively was 4.6 (0.49) mmol/L, compared to 4.9 (0.58) mmol/L (95% CI 0.45, 0.15, *p* <  0.001) among women who did not exclusively breastfeed. Among women with GDM, the reduction in fasting glucose in women who were breastfeeding was 0.22 mmol/L (95% CI 0.39, 0.05, *p* = 0.004), and in women who were not GDM, the reduction was 0.14 mmol/L (95% CI 0.37, 0.09, *p* = 0.24,). After adjustment for GDM status in pregnancy, maternal body mass index (BMI), maternal age and ethnicity, and exclusive breastfeeding was associated with a decreased fasting glucose of 0.19 (95% CI 0.318, 0.061, *p* = 0.004). After similar adjustment, there was no significant difference in triglycerides, high density lipoprotein cholesterol or low-density lipoprotein cholesterol between women who were breastfeeding and women who were not breastfeeding.

**Conclusions:**

Breastfeeding is associated with a reduction in fasting glucose levels postpartum, but not maternal lipid profile. Breastfeeding may play a role in reducing glucose intolerance in women who have had GDM.

## Background

Gestational diabetes mellitus (GDM) is increasingly common worldwide, due to changing maternal demographics, including older age and increased body mass index (BMI), and changing diagnostic criteria. Women who have GDM have an risk of type 2 diabetes mellitus (T2DM) more than 7-fold higher than women with normoglycaemic pregnancies [1]. Approximately 5% of women will develop T2DM in the first six months after a pregnancy with GDM, and a further 10% will develop diabetes 1–2 years postpartum [2]. Women with GDM are also at increased risk of cardiovascular disease (CVD) [3].

Strategies to reduce the risk of progression to T2DM in women who have had GDM is an important public health priority.

Breastfeeding may contribute the return to pre-pregnancy weight for postpartum women [4]. Information on the role of breastfeeding in reduction of progression to T2DM has been mixed, with studies both supporting and refuting this [5–7]. This protective effect was still demonstrated 30 years after the index pregnancy in a prospective cohort of women with and without GDM [8]. Meta-analysis of nine studies showed a reduction in T2DM with breastfeeding [9]. There are some methodological concerns with some of these cohorts, including retrospective reporting of breastfeeding duration and self-reporting of diabetes status [7].

In a non GDM cohort at approximately 11 years, cardiovascular differences were not seen based on duration of breastfeeding. A randomised control trial design is not suitable for trails including breastfeeding as an intervention, and so further observational data are required.

The adoption of breastfeeding in women with and without GDM may serve as a simple and potentially effective intervention in preventing the development of impaired glucose tolerance, T2DM, and CVD. As such, we aimed to explore the breastfeeding practices of women with and without GDM at six to ten weeks postpartum using data from a cohort study to evaluate their glucose tolerance and lipid profile and so provide mechanistic evidence for the demonstrated improvements in metabolic and cardiovascular outcomes.

## Methods

### Patient recruitment and sample collection

Potential participants were approached in the immediate postpartum period by a researcher. Women were eligible for this study if they had a singleton pregnancy, delivered after 37 weeks gestation, aged at least 18 years old, and could speak sufficient English to understand the study and consent process. Women were excluded if they had pre-existing type 1 or 2 diabetes, and babies with major congenital abnormalities.

One hundred and fifty-nine women with GDM and 84 women without GDM were recruited between October 2012 and May 2017, as part of a larger cohort study. Details of recruitment of women with GDM have been published previously [10]. The diagnosis of GDM was made by an oral glucose tolerance test (OGTT) at 24–28 weeks of gestation, according to the Australasian Diabetes in Pregnancy Society (ADIPS) guidelines by either a fasting venous plasma concentration of ≥5.5 mmol/L glucose and/or ≥ 8.0 mmol/L glucose 2 h after a 75 g oral glucose load [11]. The women had multidisciplinary care during the pregnancy from a team including dietitian, diabetes educator, endocrinologist and obstetrician. They were advised to follow the recommended standard of care diet for controlling blood glucose (40% carbohydrate, 15% protein and 45% fat). They undertook regular home blood glucose monitoring and commenced on insulin for raised blood sugars. Forty two percent of the women were on insulin during pregnancy. One hundred and forty-nine women without GDM in the index or previous pregnancies were recruited between December 2016 and May 2017, and were classified as not having diabetes based on the current ADIPS criteria.

Ethnicity was self-defined, and then dichotomized as Caucasian (Oceanic, European and American) and non-Caucasian (South and North-East Asian, Southern and Central Asians, and Aboriginal and Torres Strait Islander) because of increased rates of diabetes in the second group of communities. Family history was elicited of dyslipidemia, hypertension, CVD and diabetes (type 1, type 2 or GDM) in parents, grandparents and siblings.

Participants had a fasting blood test performed at six to ten weeks postpartum for blood glucose and cholesterol, triglycerides, high density lipoprotein (HDL) and low density lipoprotein (LDL) levels. Women were instructed to fast for at least eight hours. Water was permitted. The women had their weight, height and blood pressure measured. Women were asked at the time of the postpartum review about feeding practices over the last few days. They were asked if they if they were exclusively breastfeeding, exclusively formula feeding, or a mixture of methods. Breastfeeding status was categorized as exclusively breastfeeding or not exclusively breastfeeding, including women who were feeding both formula and breastfeeding.

Blood was collected according to hospital guidelines by trained phlebotomists. Analysis of results was carried out by the Austin Hospital laboratory, which acts under NATA standards for quality control.

### Statistical analysis

This study was part of a larger cohort study however sample size was calculated based on published mean fasting glucose levels postpartum in women with and without GDM [6]. Using α = 0.05 and power 0.80, the minimum sample size required in each group was 33 women.

Statistical analysis was performed using STATA 13IC (StataCorp, College Station TX, USA). Statistical significance was defined as *p* <  0.05. Continuous data were summarised as the medians and interquartile ranges and as counts and proportions for categorical variables.

The hypothesis that breastfeeding impacts postpartum glucose tolerance and lipid profiles was investigated using appropriate regression models for continuous outcomes. All models included fasting glucose, cholesterol, triglycerides, HDL cholesterol and LDL cholesterol as an input and breastfeeding status, BMI and GDM status as covariates for adjustment purposes. These variables were chosen as they are believed to independently influence glucose intolerance and abnormal lipid status. All data are presented as numbers and percentages, means and standard deviations, or medians and interquartile ranges, as appropriate.

## Results

Two hundred and forty-three women were recruited during the study period. Of these, 159 women had GDM and the remaining 84 women did not have GDM. The demographic details of the participants are described in Table 1 and are quite similar between the two groups, apart from ethnicity. Women were reviewed at a median time of 6.9 weeks postpartum (interquartile range 1.3 weeks)).

**Table 1:** Demographic details of participants

	GDM (cases)		No GDM (controls)
*n*	159		84
GDM treatment	Diet	92 (57.9%)	N/A
	Medical	67 (42.1%)	
Maternal age (years)	33.73 (31.06, 36.68)		32.33 (29.61, 34.71)
Maternal BMI at sample collection (kg/m2)	27.1 (23.8, 31.1)		26.4 (24.1, 29.1)
Maternal BMI			
Normal Weight	61 (38.6%)		27 (32.9%)
Overweight	53 (33.5%)		36 (43.9%)
Class I Obesity	21 (13.3%)		13 (15.9%)
Class II Obesity	16 (10.1%)		3 (3.7%)
Class III Obesity	7 (4.4%)		3 (3.7%)
Weight gain in pregnancy (kg)	10 (6, 13.5)		14 (10, 15.25)
Current smoker	3 (1.9%)		4 (4.8%)
Nulliparous	104 (65.4%)		45 (53.6%)
Maternal ethnicity Caucasian	84 (52.8%)		67 (79.8%)
Family history of increased cholesterol	62 (39.0%)		33 (39.3%)
Family history of hypertension	81 (50.9%)		31 (37.0%)
Family history of CVD	29 (18.2%)		18 (21.4%)
Family history of diabetes	58 (36.4%)		27 (32.1%)
Exclusive breastfeeding^a^	106 (66.67%)		65 (77.38%)

Data depicted as number (%) or median (IQR), data missing for 1 participant in the GDM group and for 2 participants in the control group for maternal BMI

Medical management for GDM included either insulin (65) or metformin (2) use

BMI classified as per WHO guidelines

^a^Defined in Methods

The rate of women exclusively breastfeeding was higher in the non-GDM group, with 77.4% of women without GDM exclusively breastfeeding and 66.7% of women with GDM exclusively breastfeeding.

The mean (SD) glucose in the group of women who breastfed exclusively was 4.6 (0.49) mmol/L, compared to 4.9 (0.58) mmol/L (95% CI 0.45, 0.15, *p* < 0.001) among women who did not exclusively breastfeed. After adjustment for GDM status in pregnancy, maternal BMI and ethnicity, exclusive breastfeeding was associated with a decreased fasting glucose of 0.19 (95% CI 0.318, 0.061, *p* = 0.004). After adjustment for BMI, age and ethnicity, women with GDM who were breastfeeding had lower fasting glucose of 0.22 (*p* = 0.12, 95% CI 0.39, 0.05) compared to women with GDM who were not breastfeeding however this difference was not seen among women without GDM (mean difference − 0.14, *p* = 0.24, 95% CI 0.37, 0.09) (Table 2).

**Table 2:** Glucose and lipids in women who were exclusively breastfeeding and women who were not exclusively breastfeeding

	Unadjusted			Adjusted difference in mean ^a^	
	Exclusively breastfeeding^b^	Non-exclusively breastfeeding			
	Mean (SD)	Mean (SD)	*p* value (95% CI)	Mean difference	*p* value (95% CI)
Fasting Glucose	4.60 (0.49)	4.90 (0.58)	< 0.0001 (−0.45, −0.15)	−0.19	0.006 (− 0.33, − 0.05)
Triglycerides	1.09 (0.96)	1.45 (0.77)	0.002 (−0.59, − 0.13)	−0.24	0.062 (− 0.49, 0.12)
LDL-Cholesterol	3.41 (0.42)	3.39 (0.83)	0.89 (−0.24, 0.27)	−0.02	0.88 (− 0.27, 0.22)
HDL-Cholesterol	1.75 (1.05)	1.57 (0.60)	0.025 (0.02, 0.33)	0.098	0.19 (−0.05, 0.24)

Units are mmol/L

^a^Adjusted for GDM, BMI, age and ethnicity

^b^Defined in Methods

There was no difference in fasting lipids between women who breastfed and those who did not, either in women who had GDM and those who do not, or the whole cohort. Women who were exclusively breastfeeding had increased HDL cholesterol, lower triglycerides, and no difference in LDL cholesterol, compared to women who were not breastfeeding. After adjustment for GDM status, BMI and maternal age there was no significant difference in triglycerides, HDL cholesterol or LDL cholesterol (Table 2).

## Discussion

Women with GDM who breastfed had a reduction in fasting glucose at six weeks postpartum compared to women who did not breastfeed. After adjustment for maternal factors, there were no changes in lipids between women who did or did not breastfeed. A similar pattern was seen in women without GDM.

Previously published data in women with and without GDM examined in the postpartum period showed similar changes in lipid status, fasting glucose, and abnormal GTT [12–14]. Specifically, in the women who breastfed, insulin levels and ratios of insulin to glucose were significantly lower, and carbohydrate utilization and total energy expenditure were higher [13].

Breastfeeding has also been shown to have no effect on total cholesterol, LDL or triglyceride levels, but increased levels of serum HDL levels after breastfeeding for 4–12 weeks when compared to a non-lactating group [15]. This protective association between lactation and increased HDL is further supported by other authors [16, 17]. Unfortunately, this protective effect of breastfeeding does not seem to continue beyond the breastfeeding period. At three years postpartum, a consistent association has not been demonstrated between breastfeeding duration and lipid levels [13] A long term effect however, has been shown for metabolic parameters, including improved insulin sensitivity in women four years after delivery, who breastfed [18].

Lactation requires mobilisation of lipids for milk synthesis [19], and may improve glucose metabolism and insulin sensitivity by increasing glucose disposal rates, enhancing lipolysis to accommodate for demands of milk production and diverting glucose to the mammary gland for utilization in milk production [20, 21]. The mediators of improved outcome may be related to prolactin level. Prolactin is involved in pancreatic structure and function, and prolactin levels in in men and women have been shown to be related to T2DM risk in a large cohort [22, 23].. We were not able to measure prolactin levels in our cohort, but this relationship between maternal prolactin and metabolic markers has been confirmed in another breastfeeding cohort [6].

It has been suggested that finding women who breastfeed at all, or for longer durations, may do so because they have an underlying better metabolic state. This view was supported by a randomised controlled trial of breastfeeding in women predominantly without gestational diabetes, in whom long term cardiovascular outcomes were not improved by duration of feeding [24]. Our data which shows more decrease in fasting glucose in women with GDM who breastfed compared to women without GDM who breastfed, contravenes this belief. It suggests that even in women with metabolic compromise, breastfeeding plays a role in prevention of long-term CVD.

This cohort adds substantial numbers of women to the previous published data regarding effects of breastfeeding on maternal metabolic status. The strengths of our study include the prospective design. We were also able to adjust for BMI and GDM status, which are well known contributors towards diabetes risk, and hence CVD risk. Breastfeeding data was reported at the time of sample collection, and so was not affected by recall bias. The prospective cohort was comprised of an ethnically diverse sample of women with GDM and thus, our findings are generalisable, within specific geographic regions, given that Asian women experience much higher rates of GDM than Caucasian women [25]. Limitations of our study include the small sample size, relatively short follow up time and the number of participants that were lost to follow up. The study aimed to determine the effects of exclusive breastfeeding, and only a small number of women were undertaking mixed feeding, so a dose response relationship could not be established. Exclusive breastfeeding was defined as exclusive feeding over the previous few days, a nonstandard definition. The criteria for diagnosis of GDM were changed between recruitment of the two cohorts, but the criteria used for recruitment of controls was more stringent, and so women who were diagnosed as non GDM using this criterion, would also have been diagnosed as non GDM using the previous criteria.

## Conclusion

We have shown a positive relationship between exclusive breastfeeding at six to ten weeks postpartum and reduction in fasting glucose levels in women with GDM. This provides further strength to the important public health message of supporting women to breastfeed for their own long-term health. Women at higher risk of long-term diabetes and CVD should receive additional encouragement and support to breastfeed.

## Data Availability

The datasets used and/or analysed during the current study are available from the corresponding author on reasonable request.
